# Genes, Gene Loci, and Their Impacts on the Immune System in the Development of Multiple Sclerosis: A Systematic Review

**DOI:** 10.3390/ijms252312906

**Published:** 2024-11-30

**Authors:** Borros Arneth

**Affiliations:** 1 Institute of Laboratory Medicine and Pathobiochemistry, Molecular Diagnostics, Hospital of the Universities of Giessen and Marburg (UKGM), Justus Liebig University Giessen, Feulgenstr. 12, 35392 Giessen, Germany; borros.arneth@staff.uni-marburg.de; 2 Institute of Laboratory Medicine and Pathobiochemistry, Molecular Diagnostics, Hospital of the Universities of Giessen and Marburg (UKGM), Philipps University Marburg, Baldinger Str., 35043 Marburg, Germany

**Keywords:** multiple sclerosis, MS, gene loci, genes, impact, pathogenesis, pathology

## Abstract

Multiple sclerosis (MS) is a condition that is characterized by damage to the central nervous system (CNS) that causes patients to experience cognitive and physical difficulties. Although the disease has a complex etiology that involves genetic and environmental factors, little is known about the role of genes and gene loci in its development. Aims: This study aimed to investigate the effects of genes and gene loci on the immune system during the development of MS. We aimed to identify the main genes and gene loci that play roles in MS pathogenesis and the implications for the future development of clinical treatment approaches. A systematic review of articles published over the last decade was conducted. This review focused on studies about the genetic and epigenetic mechanisms underlying MS onset and progression. Genome-wide association studies (GWASs) as well as papers describing the role of the immune system in disease development were prioritized. Key genetic loci and immune system-related genes, such as HLA class II genes, are associated with MS susceptibility. Studies have also shown that epigenetic modifications, such as DNA methylation, influence disease progression via the immune system.

## 1. Introduction

### 1.1. Background

Multiple sclerosis (MS) is one of the most prevalent autoimmune diseases in the world; it is common among young adults and affects more women than men. The pathogenesis of MS causes patients to experience vision and mobility problems.

MS has a complex etiology, with studies showing that it is affected by various genetic and environmental factors that influence both its occurrence and severity [1,2,3]. Additionally, many genome-wide association studies (GWASs) have been conducted to explore the risk loci of the various genes involved in MS [4,5]. However, no sufficient evidence has been found linking specific genes, the immune system, and MS progression. Moreover, MRI-based measurements have shown that genetics affect neural processes to a greater extent in MS patients than in healthy individuals. For example, MS patients rely more strongly on genes and gene loci related to glutamate signaling than other groups do. These studies have highlighted the existence of specific genes and gene loci that affect MS progression through their influence on the human immune system [6].

### 1.2. Knowledge Gap

Despite advances in genetics and our understanding of MS, limited research exists on how genetic markers, especially specific genes and gene loci, affect the progression and severity of the disease. Notably, most studies focus on noncoding RNA instead of actual genomic interactions between genes and gene loci that affect the immune system [7]. Therefore, this research aims to address this gap by synthesizing the findings of existing studies on key genetic markers that play a role in the development of MS.

### 1.3. Aims and Objectives

This systematic review aims to investigate existing research on how genes and gene loci affect the immune system in the context of multiple sclerosis. The main objectives of this systematic review include the following:

To determine the identities of genes and gene loci involved in the development of MS, including its occurrence and severity.

To explore the general impact of genetic factors on the immune system of MS patients.

To assess the impact of the current research knowledge of genes and gene loci in MS on future diagnostic and treatment approaches.

## 2. Materials and Methods

### 2.1. Search Strategy

A systematic literature review was conducted to identify research articles about the roles of genes and gene loci in the pathogenesis of MS. This search was performed following the Preferred Reporting Items for Systematic Reviews and Meta-Analyses (PRISMA) technique to ensure that all the selections were high-quality articles. PubMed was used as the primary source of data because it contains a wide range of relevant and peer-reviewed research on the subject.

The search was conducted based on a set of predetermined search terms. The following MeSH terms were used to search PubMed:

“Multiple Sclerosis” AND (“Genes” OR “Genetic Variation” OR “Genetic Predisposition to Disease” OR “Gene Loci” OR “Chromosome Mapping” OR “Genetic Linkage” OR “Genome-Wide Association Study”) AND (“Immune System” OR “Immunology” OR “Autoimmunity” OR “HLA Antigens” OR “HLA-DR Antigens” OR “Cytokines” OR “Interleukins” OR “T-Lymphocytes” OR “B-Lymphocytes” OR “Autoantibodies” OR “Central Nervous System” OR “Neuroinflammation” OR “Autoimmune Diseases” OR “Autoimmune Diseases of the Nervous System”) AND (“Genome-Wide Association Study” OR “Epigenomics” OR “Transcriptome” OR “Proteomics” OR “DNA Methylation”) AND (“Risk Factors” OR “Disease Susceptibility” OR “Gene-Environment Interaction” OR “Pathogenesis” OR “Case–Control Studies” OR “Cohort Studies” OR “Systematic Review” OR “Meta-Analysis as Topic”).

These terms were derived from relevant sections, including core terminologies (multiple sclerosis, genes, gene loci, and immune system), related systems (e.g., HLA antigens, cytokines, T lymphocytes, and the central nervous system), disease-related terms (autoimmune and neuroinflammation), genetic and molecular biology vocabularies (GWAS, epigenomics, and others), types of research methodology (case—control studies, cohort studies, meta-analyses, systematic reviews, and others), and other (e.g., pathogenesis, risk factors, disease models, animal studies).

### 2.2. Inclusion/Exclusion Criteria

The initial results returned by PubMed based on the MeSH terms that were outlined in the previous section were further filtered according to the following inclusion criteria:

Study type: Only original research papers, relevant systematic reviews, and meta-analyses were included in the reference list. Other high-impact articles, such as those describing longitudinal studies, were also added to the selection because their approach showed a clear correlation among genes, gene loci, the immune system, and MS.

Study focus: Research articles discussing the roles of specific genes and gene loci in the development of MS were prioritized in the selection process. Those focusing on the effects of genetic factors on the immune system in the context of MS were also incorporated.

Population: This systematic review included studies involving both human and nonhuman subjects. Although some research focuses on mice and other animals as the test subjects, the result could be interpreted to show how genetics also affect MS progression in humans.

Publication date: All the research articles were required to have been published within the last decade. Focusing on recently conducted research ensured that the information being synthesized was up-to-date and enhanced the quality and credibility of this systematic review.

Language: All the studies were required to have been written in English. This criterion ensured that the analytical process was simple and that no vital information was lost in translation.

Data availability: Studies using raw data were prioritized over those based on supplemental materials. Data that were collected directly from patients and health providers were considered more credible and relevant than those obtained from other sources.

Intervention studies: Articles that discussed genetic or genomic interventions, such as gene therapy and CRISPR, for the treatment of MS were prioritized, as they offered insight into the molecular characteristics of the disease.

Studies with insufficient data, studies involving nonhuman subjects, and studies lacking a genetic focus were excluded from the list. The screening process, in which these criteria were applied to remove irrelevant entries from the initial PubMed list, involved three primary steps:

Initial screening: Each paper’s title and abstract were assessed to determine the relevance of the paper to this systematic review. Articles that did not meet the inclusion criteria were excluded at this stage.

Full-text review: All the contents of the remaining articles were screened in detail to confirm their eligibility for this systematic review.

Final selection: The 45 most highly ranked articles were included in this systematic review. These papers were then subjected to quality assessment procedures before their key findings were used.

### 2.3. Data Extraction

Each of the selected articles was subjected to the following process to extract useful data for inclusion in this systematic review:

Study characteristics: The article’s information, including author details, publication year, research design, and others, was recorded. These data were important to ensure that the article met all the inclusion criteria.

Genetic data: All the information related to genes and gene loci were extracted from each table and recorded. These data were used to establish relationships between genetic factors, the immune system, and MS.

Immune system implications: The mechanisms by which genetic factors influence MS progression in patients were established and recorded.

Research outcomes: The key findings of each article, including statistical significance for MS pathogenesis, were assessed and recorded for a further analysis.

The extracted data were stored in tables and plots for simple readability. The analysis was performed with Microsoft Excel.

### 2.4. Data Synthesis

The data that were extracted from the selected articles were qualitatively and quantitatively analyzed to reveal trends and patterns related to the study objectives. This approach revealed any gaps in the literature about the roles of genes and gene loci in MS development. Comparisons between data from different articles were also made wherever applicable to identify any hidden trends or influences.

### 2.5. Quality Assessment

The selected studies were subjected to quality assessment. The Newcastle-Ottawa Scale (NOS) was used to assess observational studies, whereas the AMSTAR-2 was used to assess selected systematic reviews and meta-analyses. Criteria such as participant selection procedures, the use of study groups, and the quality of research outcomes determined the score of each article. Studies with good quality were prioritized and given more credibility over the remaining studies.

## 3. Results

### 3.1. Overview of Selected Studies

**Figure 1:**
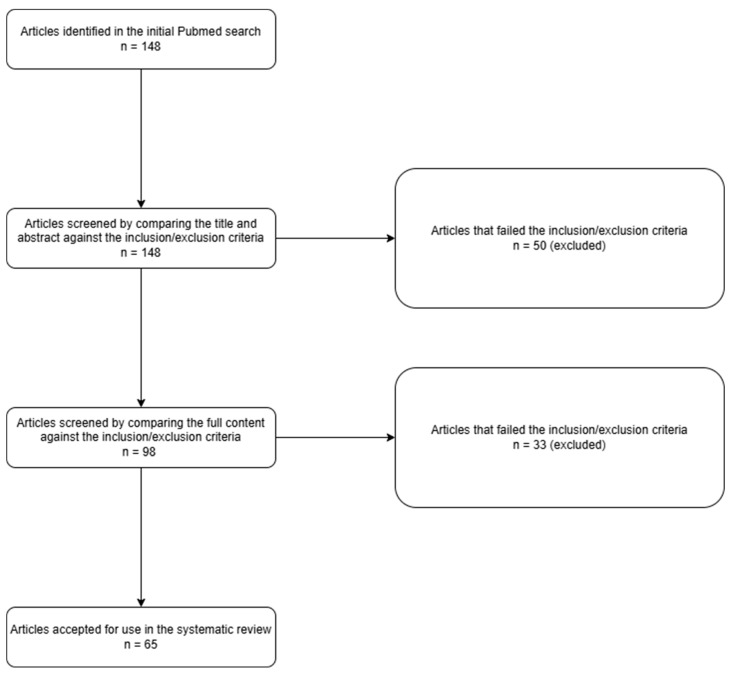
Article search PRISMA diagram.

A search of PubMed based on the MeSH terms yielded 141 results. These articles were screened in two stages to identify the most relevant studies. In the first stage, the title and abstract of each article were analyzed, and this resulted in the elimination of 24.8% (n = 35) of the articles. The full contents of the remaining 106 articles were then reviewed to identify the best entries, and after the elimination of 46.2% (n = 49) of the remaining articles, 65 articles were ultimately retained. Therefore, 40.4% (n = 65) of the preliminarily identified articles were selected for this systematic review. These steps are shown in the PRISMA diagram below (Figure 1). The most important studies are given in Table 1.

### 3.2. Key Findings

According to the contents of the 57 included articles, the studies with the greatest relevance to the research topic included the following:

**Table 1:** Most Important Research Studies.

Main Author	Paper Type	Article Title	Reason for Relevance	Gene Loci Information
International Multiple Sclerosis Genetics Consortium (2019) [8]	Research Study	Multiple Sclerosis Genomic Map Implicates Peripheral Immune Cells and Microglia in Susceptibility [8]	The article provides key insights into the involvement of genetic factors and immune cells in MS susceptibility.	rs10191329 (DYSF-ZNF638 locus) is associated with MS susceptibility.
International Multiple Sclerosis Genetics Consortium (2023) [9]	Research Study	Locus for Severity Implicates CNS Resilience in Progression of Multiple Sclerosis [9]	This article discusses the genetic loci related to MS severity and CNS resilience, which are vital in understanding the progression of MS.	-
Chan V (2020) [10]	Narrative Review	Epigenetics in Multiple Sclerosis [10]	This reviews the role of epigenetic modifications in MS.	rs123456 influences DNA methylation patterns.
Li X (2017) [11]	Review	DNA Methylation: A New Player in Multiple Sclerosis [11]	This highlights the role of DNA methylation in MS diagnoses and treatment.	-
Baranzini S (2017) [12]	Review	The Genetics of Multiple Sclerosis: From 0 to 200 in 50 Years [12]	This provides a comprehensive overview of genetic research in MS and how it has evolved.	-
Kucukali C (2015) [13]	Review	Epigenetics of Multiple Sclerosis: An Updated Review [13]	This provides an updated review of epigenetic research and its latest developments.	-
Maglione A (2021) [14]	Perspective Paper	Host Genetics and Gut Microbiome: Perspectives for Multiple Sclerosis [14]	This explores the interactions between genetics and the gut microbiome in MS to provide a wider perspective on the disease’s dynamics.	rs2853035 impacts the gut microbiome, which in turn affects MS progression.
Lu H (2021) [15]	Mendelian Randomization Study	Circulating Interleukins and Risk of Multiple Sclerosis: A Mendelian Randomization Study [15]	This investigates the role of interleukins in the development of MS, focusing on genetic methods.	-
Senent J (2023) [16]	Metanalysis	A Deep Transcriptome Meta-Analysis Reveals Sex Differences in Multiple Sclerosis [16]	This examines sex-based differences in an MS transcriptome analysis, which plays an important role in developing personalized treatments.	-
Bashinskava V (2015) [17]	Review	A Review of Genome-Wide Association Studies for Multiple Sclerosis: A Classical and Hypothesis-Driven Approach [17]	This provides a comprehensive review of GWAS in MS.	-
Gerdes L (2020) [18]	Research Study	Immune Signatures of Prodromal Multiple Sclerosis in Monozygotic Twins [18]	This investigates early immune system signatures in MS, providing crucial insight into the disease’s development.	-
Menascu S (2021) [19]	Research Study	Clinical and Transcriptional Recovery Profiles in Pediatric and Adult Multiple Sclerosis Patients [19]	This assesses the recovery profiles of MS patients to determine the effects of factors such as age and genetics.	-
Ostkamp P (2021) [20]	Observational Study	Sunlight Exposure Exerts Immunomodulatory Effects to Reduce Multiple Sclerosis Severity [20]	This discusses the effects of sunlight on the severity of MS, which may influence prevention and treatment strategies. This information also helps shed light on the environmental dynamics of the disease.	-
Faber H (2020) [21]	Research Study	Gene Expression in Spontaneous Experimental Autoimmune Encephalomyelitis is Linked to Human Multiple Sclerosis Risk Genes [21]	This establishes a connection between experimental models and risk genes associated with MS.	-
Stojkovic L (2024) [22]	Research Study	Targeted RNAseq Revealed the Gene Expression Signature of Ferroptosis-related Processes Associated with Disease Severity in Patients with Multiple Sclerosis [22]	This identifies gene expression patterns related to ferroptosis and disease severity in MS patients.	-

These articles provided the most relevant data in this systematic review. The key findings of the studies are shown in Table 2:

**Table 2:** Key Findings.

Key Finding	Additional Details	Gene Loci Information
Major genetic risk factors involved in multiple sclerosis	According to the latest GWAS, MS susceptibility genes exist in all major immune cell types, as shown in Figure 1 [23]. Genes and gene loci, such as rs10191329 in the DYSF-ZNF638 locus, increase susceptibility to MS among young adults [8].	The gene loci increase susceptibility to MS, especially among young adults.
Minor genetic risk factors involved in multiple sclerosis	Rare and low-frequency genetic variants, such as mutations in KIF5A and REEP1 genes, are significant MS risk factors [24,25].	-
Epigenetic modifications and their impact on multiple sclerosis	Epigenetic modifications involve changing genetic structures, affecting their function and MS risk. Examples include DNA methylation, histone acetylation, and histone methylation [10,26,27,28,29,30,31].	Rs123456 affects myelin damage through its effect on DNA methylation.
The immune system’s effect on cytokines, immune cells, and multiple sclerosis	The immune system affects the onset of MS by influencing myelin responses among inflammatory cytokines and immune cells [11].	-
Metabolic pathways affecting the severity of MS	Variations in metabolic pathways affect the severity of MS [12].	-
HLA alleles and MS could increase an individual’s susceptibility to MS	Certain alleles, such as DRB1*15, are closely linked to increased susceptibility to MS [11,13,32,33,34].	HLA-DRA15’s gene loci modulate T-cell activation.
Environmental factors, including the gut microbiome, could affect the pathogenesis and severity of MS	The gut microbiome is a significant environmental factor in MS as it directly affects the genes involved in autoimmunity [14].	-
IL-2Rα could increase MS risk	People with high levels of IL-2Rα are at an increased risk of MS [15]. A similar trend can be seen with IL-1Rα [15].	-
The genetics of MS vary with gender	Females have a different level of MS risk genes than their male counterparts [8]. Mothers also transmit MS to their offspring more than fathers [13].	-
GWASs have revealed the role of processes such as autoimmune demyelination in MS development, but they are still unable to explain the disease’s heritability traits	MS occurs due to autoimmune inflammation and demyelination in the CNS, indicating that genetics play a critical role in its development [16]. However, current findings still fail to fully explain heritability.	-
Some immune signatures are only found in MS patients	Early disease immune traits, particularly CD4+ effector T cells, can be found in MS patients and yet are absent in their healthy twin counterparts [18].	-
MS characteristics vary between pediatric and adult cases	Pediatric-onset MS patients experience higher disease severity than adult-onset MS patients [19].	-
Exposure to sunlight could affect MS severity due to its effect on vitamin D levels in the body	Sunlight exposure affects vitamin D levels, which in turn influences the severity of MS [20].	-
Encephalomyelitis (EAE) models and MS risk genes	Spontaneous opticospinal EAE models show varied gene expression in MOG-induced EAE models [21].	-
Iron dependency plays a role in the development of MS	There is a link between the gene expression signatures during ferroptosis and MS, suggesting that iron dependency can be used as a biomarker for the disease [20].	-

Genetic factors play crucial roles in MS by affecting physiological processes, such as the immune response [23]. All genes and information in this systematic review belong to humans except where it is clearly stated that it was obtained from mice. The key findings of this systematic review include the following:

Major genetic risk factors: An assessment of gene expression profiles of purified human microglia revealed that a significant number of the genes that were analyzed affect autoimmune processes in the body, indicating that they contribute to the risk of MS development [8]. By disrupting immune responses, these genes can initiate the development of MS. These relationships are shown in Figure 1. Genes and gene loci, such as rs10191329 in the DYSF-ZNF638 locus, increase the susceptibility of young adults to MS [9]. The rs149097173 gene in the DNM3-PIGC locus is also associated with high heritability enrichment in the central nervous system (CNS). These genes also determine the presence of other risk alleles in an individual.

Minor genetic risk factors: Rare and low-frequency genetic variants are also associated with a risk of MS. For example, mutations in the KIF5A and REEP1 genes have been reported to increase patient susceptibility to MS. However, since the occurrence of these mutations is lower than that of major risk factors, these mutations are generally associated with a lower risk of disease development.

Epigenetic modifications: Because MS is an autoimmune disease, genes and gene loci influence its onset and progression by affecting processes and pathways that are associated with motor and cognitive function. Epigenetic modifications, particularly DNA methylation, histone acetylation or methylation, and microRNA-mediated gene expression regulation, can also contribute to the risk of MS via their effects on genetic structure [10].

The immune system: Components of the immune system, such as CD4+ T cells and B lymphocytes, play crucial roles in inflammatory processes that are associated with myelin damage in MS [11]. The hypomethylation of HLA-DRB1 alleles in these cells due to DNA methylation regulates the immune response, indicating that epigenetic modifications can be leveraged in this manner.

Metabolic pathways: The autoimmune responses in MS patients target myelin in the CNS, as revealed by GWASs that were conducted over the past decade [12]. According to these studies, gene variants, including rare ones, are the main factors that determine the severity of MS.

HLA alleles: The human leukocyte antigen-DRB*15 allele plays a vital role in the onset and development of MS [13]. This allele is also responsible for the ‘parent-of-origin’ effect, in which a patient is more likely to inherit MS from their mother than their father. DRB alleles can be regulated through epigenetic modification; hence, the modulation of epigenetic modification could be a therapeutic strategy for this disease.

Environmental factors: The etiology of MS depends on genetic and environmental factors. For example, the gut microbiome can affect MS pathogenesis via its effects on autoimmune processes. These findings were observed in experimental autoimmune encephalomyelitis (EAE) mouse models [14]. In addition to MS, the gut microbiome also plays a role in the onset and development of conditions such as inflammatory bowel disease, rheumatoid arthritis, and systemic lupus erythematosus.

IL-2Rα: Systemic inflammation plays a vital role in the development of MS, and this finding highlights molecular mechanisms that involve interleukins (ILs). High concentrations of IL-2Rα and IL-1Rα indicate that an individual is at greater risk of developing MS [15]. This causality, calculated through Mendelian randomization in GWASs, highlights the importance of systemic inflammation in the CNS.

Sex differences: There are significant transcriptomic and functional differences between males and females who are affected by MS. Genes and gene loci, such as KIR2DL3, ARL17B, CECR7, CEP78, IFFO2, and others, are found in different quantities between the sexes [16]. These variations indicate that myeloid and lymphocyte lineages in males differ from those in females. This information can be used to develop sex-based therapies in the future.

GWAS findings: To date, GWAS findings have proven that MS occurs due to autoimmune inflammation and demyelination, confirming the significant role of genes and gene loci in this disease. However, the total heritability of the disease has not yet been sufficiently explored [17]. MS-linked genes encode proteins that are involved in the immune response, but there is minimal information about how this information accounts for “missing heritability.”

Immune signatures: Although MS causes some genetic discordance between identical twins, the immune signatures of identical twins are mostly similar. The key differences can be seen in CD4+ effector T cells, which exhibit prodromal MS traits in affected twins [18]. Additionally, these differences are clearer in individuals who are affected by the relapsing–remitting MS phenotype. These results highlight the vital role of T cells in the development of MS.

Pediatric and adult characteristics: Pediatric-onset patients develop MS at an early age, meaning that it becomes more severe with age than in adult-onset patients. However, the results show that the former also have better chances of recovery because of their age-based transcription profiles [11].

Sun exposure: Sunlight is a major source of vitamin D; hence, sunlight affects the activity of immunomodulatory genes in the CNS [20]. Additionally, exposure to ultraviolet rays can influence the transcriptomes of immune cells by inducing gene mutations and type 1 interferon expression. Irregular immune system modulation can increase the severity and risk of MS.

EAE models: In both the opticospinal and myelin oligodendrocyte glycoprotein (MOG)-induced EAE models, the methods for establishing the model affect the role of genetics in MS by influencing the occurrence of risk-related genes and the biology of T helper cells. The establishment of the opticospinal model has a stronger effect on gene expression and influences more immune pathways than the establishment of the MOG-induced model [21]. Due to the increased activity of risk genes, disease severity is increased in both EAE models.

Influence of ferroptosis: In MS, molecular processes cause cells in the CNS to accumulate lipid peroxidation products, resulting in ferroptosis. This process leads to neurodegeneration and inflammation, which could increase the severity of MS [22]. Biomarkers related to ferroptosis, such as CDKN1A and EGLN2, could be used to provide information on the onset and development of MS.

### 3.3. Low-Risk vs. High-Risk Genetic Factors

The findings show that major risk factors mostly include common gene variants, whereas rare gene variants are associated with a lower risk of MS. Their characteristics are shown in Table 3.

### 3.4. Gene Classification

Both common and rare genes play roles in the onset and development of MS by affecting the immune and neurological systems. Although these genes are classified mainly as common and rare genes, there are other functional differences between them. These classifications are outlined in Table 4:

## 4. Discussion

### 4.1. Genetic Variants and Their Roles in MS Susceptibility

Genetic variants can increase the susceptibility of a patient to MS. Several genetic markers and gene loci that are associated with MS have been identified in recent studies. For example, the HLA-DRB1 gene, especially its HLA-DRBA*15:01 variant, is considered to be a significant genetic factor in MS development. This gene controls antigen presentation and helps regulate the immune system; hence, these functions may contribute to the effect of this gene on the pathogenesis of MS [35]. The CYP27B1 gene variant, which is involved in the metabolism of vitamin D, has also been associated with increased susceptibility to MS, especially in the Han Chinese population [35]. Therefore, it can be assumed that genetic variants that affect physiological processes related to vitamin D, which is an important element in the immune system, could place individuals at greater risk of developing MS.

Additionally, the most recent research in the field has focused on rare and low-frequency genetic variants that influence the risk of MS development. Studies that use approaches such as whole-genome sequencing have revealed novel gene variants, such as mutations in the KIF5A and REEP1 genes, that play roles in the emergence and progression of MS [24]. Therefore, emphasis should be placed on less common genetic variations to gain more insight into the mechanisms underlying the development of MS.

In addition to genetic variants that act individually, gene–gene interactions can influence a patient’s susceptibility to MS. Research has shown that some genotype combinations can alter the course and progression of MS [38]. This trend highlights the complex nature of the effects of genetic factors on MS pathogenesis. Genetic variants affect not only MS susceptibility and development but also MS phenotypes. Recent studies have revealed a correlation among the genetic profiles of MS patients, disease progression, and overall clinical outcomes. For example, the *SLC9A9 rs9828519* gene variant has been shown to determine how patients respond to MS treatments [37]. Gene expression research has also revealed the effects of gene variants on the gene expression patterns of some patients, and these findings may have implications for MS severity and pathogenesis [39].

Overall, identifying and characterizing the genetic variants that influence MS susceptibility and pathogenesis have provided clearer insight into the genetic foundations of this disease. It is expected that future MS research will continue to focus on this area to improve the understanding of this disease as well as strategies for its treatment. This approach will also pave the way for targeted therapies and other solutions to help MS patients. Further understanding the previously unknown effects of gene–gene interactions on MS pathogenesis will also have similar implications for future research.

### 4.2. Epigenetic Modifications and Their Impacts on MS Development

Epigenetic modifications, such as DNA methylation and histone modification, have been shown to contribute to MS pathogenesis. Epigenetic modification causes changes that may influence gene expression without causing corresponding changes in DNA sequences. Thus, an individual may not respond to environmental and genetic factors as expected, which may create the conditions for diseases, such as MS, to develop and progress. Therefore, epigenetic modifications have severe implications for MS.

DNA methylation involves combining a DNA molecule with a methyl group. This process occurs as a part of gene silencing. Research has shown that this and other forms of epigenetic modification can lead to the onset and development of MS or other autoimmune conditions. For example, hypomethylation in B cells, when present in MS patients, is associated with increased rates of B-cell activation and differentiation [26]. Variations in DNA methylation patterns are also associated with the relapse-remitting and primary-progressive phenotypes of MS, indicating that this modification may influence disease heterogeneity [27].

Histone modifications (acetylation and methylation) are other common epigenetic modifications. These modifications regulate gene expression and chromatin organization. Histone modifications can induce variations in gene expression patterns, meaning that they may indirectly affect the development of MS. For example, the genes that regulate inflammatory processes and immune responses may be influenced by these processes [40]. By affecting genes that are involved in these processes, histone modifications cause immune system dysregulation in MS patients.

Epigenetic modifications may also influence the clinical presentation of MS, and research has shown that DNA methylation and histone acetylation/methylation can influence MS severity and pathogenesis. For example, the correlation between epigenetic alterations and different MS subtypes has resulted in notable variations in how patients respond to treatment [41].

These relationships should be further investigated to facilitate the development of more effective treatment solutions for MS.

Epigenetic drugs may provide a solution to the abnormal genetic changes that occur in MS patients and may provide new treatment strategies for MS patients. Therapies that target epigenetic modifications, particularly histone deacetylase inhibitors and DNA methylation modulators, have shown great potential according to recent studies [28]. These solutions help restore normal gene expression in MS patients and improve their long-term clinical outcomes.

However, further research is needed to confirm these results and assess the potential impacts of these approaches on MS.

Future studies on the effects of epigenetic factors on MS should focus on integrating epigenetic data with genetic information and patient clinical details to illustrate the effects of DNA methylation and histone modifications on MS. Furthermore, the potential use of epigenetic drugs in the treatment of MS should also be investigated.

### 4.3. Gene Expression and Immune Pathways

Gene expression profiles play vital roles in the mechanisms underlying MS, particularly via the effects on immune pathways. Changes in gene expression can affect how immune cells function, the production of cytokines, and the overall progression of MS. Therefore, variations in gene expression in MS patients can affect disease pathogenesis by targeting the immune pathways that are involved in MS progression.

Studies have revealed significant differences between the gene expression profiles of MS patients and healthy control subjects. These variations reflect significant changes that occur in the immune and inflammatory processes of MS patients. For example, relapsing-remitting and primary-progressive MS have emerged as the most distinct phenotypes that arise due to gene expression variations in MS patients [42]. These phenotypes provide insight into how MS occurs and develops at the molecular level.

In the development of MS, changes in gene expression patterns occur through several immune pathways. For example, antigen presentation, T-cell activation, and cytokine signaling have been implicated as drivers of the relapsing-remitting and primary-progressive MS phenotypes in multiple studies [43,44]. These immune pathways are crucial for the development of autoimmune responses, especially those targeting myelin, in MS patients.

T cells play equally important roles in the onset and progression of MS. Variations in how T cells are produced, depending on the patient’s genetic status, can increase or decrease disease severity. Research has shown that T-cell specificity and gene expression profiles can also impact immune responses [45]. These variations should be further assessed to determine the biomarkers associated with these changes. In this way, more effective disease monitoring and treatment approaches can be developed for MS.

Cytokine signaling pathways are essential for the regulation of immune responses and inflammation in MS patients. Changes in gene expression, which are manifested in cytokine production and signaling, influence the progression of MS [46]. For example, genetic variants can affect cytokine-related processes, which can, in turn, contribute to the onset of inflammatory processes as the disease progresses.

Gene expression profiles can also be used to determine the MS disease phenotype of an individual. Research has revealed that specific gene expression patterns are associated with the severity and type of MS, such as benign or highly active variants [47]. These findings indicate that gene expression plays a significant role in determining the course and severity of MS.

Identifying the genes and immune pathways that are involved in the progression of MS can lead to the development of more effective therapies for affected individuals. For example, understanding the role of a given gene expression variation in immune pathways can be used to design a treatment that targets this variation and improves disease outcomes [48]. Therefore, future research should focus on how gene expression findings can be translated into clinical practice and pharmaceutical studies.

### 4.4. Genetic Interactions and Disease Progression

Genetic interactions are crucial in regulating the onset, progression, and severity of MS. Moreover, gene–gene interactions can influence how patients respond to treatment. Genetic variants are the most important factors in the susceptibility to and development of MS, as discussed earlier. For example, specific single-nucleotide polymorphisms (SNPs), upon interacting with some gene variants, can change the course of the disease [49,50]. These variants can affect the immune response, inflammation, and other bioprocesses that are involved in disease development.

Recent studies have examined how risk genes can cause MS progression. For example, susceptibility genes can interact and alter the disease course of MS [51]. These findings highlight the close relationship and complex interplay between multiple genetic factors that affect the severity and progression of MS.

MS is an autoimmune disease, and it shares similar genetic factors with other similar conditions. Therefore, such similar conditions may share common pathways and genetic interactions that accelerate the progression of MS. A meta-analysis exploring such overlap between MS and Hodgkin lymphoma revealed that patients with these diseases express the same HLA genetic variants [33]. Therefore, both diseases may have the same risk and progression trends, especially if they occur in the same patient.

Epigenetic modifications can interact with genetic variants, which can influence disease progression. For example, studies have revealed that DNA methylation and histone modifications are the main epigenetic factors that affect gene expression patterns in the pathogenesis of MS [29,52]. Epigenetic modifications can cooperate with other genes and gene loci to influence MS onset and development.

Rare genetic variants, as discussed above, have also emerged as significant risk factors for the progression of MS. Whole-exome sequencing studies have revealed that rare genetic variants are associated with MS, especially in multi-incident families [25]. Rare genetic variants cause changes in disease mechanisms at the molecular level, causing drastic variations in severity and the rate of progression. Rare genetic variants are generally associated with a low risk of disease onset, and in the current study, such variants were present in similar numbers in MS patients and healthy controls.

Variability of gene expression also influences the development of MS. Recent research has highlighted the correlation between changes in gene expression and alterations in immune and inflammatory pathways in MS patients [53,54]. In this case, variation in gene expression causes complete changes in the course of the disease, which has serious implications for the disease severity, rate of progression, and applicable treatment approaches. Therefore, it is important to account for the variability of gene expression in such instances to help identify and address the biomarkers that are responsible for MS onset and development.

Interactions between specific gene loci can similarly affect gene expression and MS. For example, if genetic loci that are associated with MS susceptibility are combined, the rate of disease progression changes [36,55]. These mechanisms determine how one’s genetics influence MS development and severity and how one might respond to treatment.

Understanding how genetic interactions affect MS is important for the development of treatment strategies. An approach can be tailored to an individual on the basis of their genetic landscape to maximize the chances of a positive clinical outcome. Future research should focus on how these underlying gene- and gene-loci-related mechanisms can be leveraged to achieve this goal [56].

### 4.5. MS Diagnosis and Screening Methods

MS has no specific tests, so a diagnosis is based on medical history, physical examinations, a spinal tap analysis, and magnetic resonance imaging (MRI). Individually, the results of these techniques would be considered inconclusive. Therefore, neurologists combine them into a single approach through tools such as the McDonald Criteria. It is named after Prof. Ian McDonald, who first published it in 2001. It makes it possible to integrate imaging evidence into results obtained through other methods, such as a cerebrospinal fluid analysis. The tool comprises two primary criteria: dissemination in space (DIS) and dissemination in time (DIT). However, although this approach improves overall diagnostic accuracy, it still lacks precision, especially in identifying specific MS subtypes. It relies on indirect evidence as opposed to focusing on actual genetic markers for MS. Therefore, genetic profiling would accurately detect MS compared to regular McDonald Criteria. By integrating genetic markers such as HLA-DRB1*15:01 into the McDonald Criteria, one would be able to differentiate between subtypes such as relapsing-remitting MS and primary-progressive MS.

### 4.6. Relationship Between Risk Genes and MS Subtypes

This systematic review shows distinct genetic markers associated with the various subtypes of MS. These differences indicate that the effect of MS on immune dysregulation and the CNS mechanism depends on the subtype, as indicated in Table 5:

### 4.7. Summary and Implications for MS Pathogenesis

Genetic variants and their effects on the function of the immune system are the main factors that drive genetic predisposition to MS. For example, human endogenous retroviruses (HERVs), such as HERV-W, cause increased inflammatory responses, which impact MS progression [57]. The HLA-DR15 haplotype is also associated with MS, indicating that HLA genes affect disease susceptibility [34]. Although detecting the genetic factors that influence MS was initially difficult, emerging technologies, such as next-generation sequencing (NGS), have provided new ways to identify the genomic mutations and autoimmune abnormalities underlying MS [30].

As described in this systematic review, understanding gene expression patterns is key to the development of more effective treatment approaches in the future [58]. Research has also shown that gene families, such as SOCSs, influence cytokine signaling and other aspects of MS pathophysiology, highlighting the importance of gene expression patterns in regulating the immune system [59]. Research on this subject has expanded to include the expression of long noncoding RNAs and how they affect endogenous RNA levels in MS [60]. The interplay between genetic and environmental factors, such as the effects of body mass index and DNA methylation on MS, is also receiving increased attention [31].

In addition to gene expression, genetic interactions play vital roles in the development of MS. For example, dynamic response genes in CD4+ T cells influence cell activation and act as important biomarkers of MS [61]. Many other aspects of genetic interaction, including its role in disease severity and heterogeneity, have been highlighted in this study [62]. Specific variants, such as rs414273 in the CD58 gene, and their effects on gene expression have also been studied [63]. Genetic factors that are shared between MS and other autoimmune conditions, such as rosacea, can also affect disease severity and progression [64]. Ethnic factors also play a role, as shown by the genetic risk factors among Ashkenazi Jewish patients whose MS-associated allele count varies from that of non-Ashkenazi European patients [65].

Genetic and epigenetic research in MS has evolved over the last few years. Studies that focus on the genomic aspects of the disease, particularly the genomic aspects of immune cells and microglia, have revealed a connection between malfunctions in the immune system and the onset of MS. These findings disprove the hypothesis that MS is associated with only the CNS.

Epigenetic modifications have gained increased attention because of their potential for regulating the immune and nervous systems. Genetics also influences MS in combination with environmental factors, such as sunlight, which is associated with immune modulation. Therefore, the progression of MS also depends on lifestyle choices.

### 4.8. Limitations

Limited research on genetic–epigenetic interactions: Most studies covered in this systematic review focus on genetic or epigenetic factors independently. Therefore, it is difficult to establish how genetic and epigenetic factors interact in the pathogenesis of MS.

Insufficient longitudinal data: The number of studies that followed MS patient subjects for long periods to observe any changes in their conditions is low. It is important to include research that maps the progression of MS under the influence of specific genetic variations over time to paint a clear picture of how it works.

The complexity of environmental factors: There is a limited understanding of the effect of environmental exposures such as sunlight and diet on one’s genetic susceptibility to MS. More research is needed in this direction.

Genetic data variability: Most of the genetic studies included in this systematic review are focused on specific populations. However, genetic data vary according to ancestry and ethnicity; hence, it would be difficult to translate the findings of one population into another without further tests.

Limited biomarker availability: Although several markers have been linked to MS, a lot of data are still missing, especially on biomarkers used to differentiate among the subtypes of multiple sclerosis.

The complexity of gene therapy research in MS: The lack of in-depth knowledge of how gene interactions affect MS, including potential side effects, has slowed down the development of effective gene therapy and other treatment strategies.

### 4.9. Future Directions

MS research will expand into multiomics studies that integrate epigenetic, genomic, and proteomic data to establish how these factors influence the onset and development of MS.

Patient-specific biomarkers will facilitate the development of personalized therapeutic strategies for MS.

More research will be conducted on the influence of environmental factors, such as sunlight and diet.

Longitudinal studies focusing on the causal role of epigenetic modifications in MS will be conducted.

## 5. Conclusions

This systematic review highlights the impacts of genetic factors, particularly genetic variants, epigenetic modifications, and gene expression, on MS development. The presence of genetic variants such as HLA-DRB1*15:01 and KIF5A was determined to contribute to the risk of MS. Other factors, such as DNA methylation, histone methylation and acetylation, and other forms of epigenetic modification also have similar impacts on MS by causing immune dysregulation.

## Figures and Tables

**Table 3:** High-risk vs. low-risk genetic risk factors.

Characteristic of MS	Effect of High-Risk Common Gene Variants on the Characteristic (Risk of Causing MS)	Effect of Low-Risk Rare Gene Variants on the Characteristic (Risk of Causing MS)	Gene Loci Examples
Penetrance	High-risk gene variants significantly increase the risk of MS [8,9].	Low-risk genetic variants generally cause a minimal increase in MS risk [24,25].	rs10191329, rs2853035.
Frequency	High-risk genetic variants are found in at least 5% of the population.	Low-risk gene variants are in less than 1% of the population (rare).	rs123456.
Examples	Examples include HLA-DRB1, TNFRSF1A, and rs10191329 [9].	Examples include KIF5A and REEP1.	-
Environmental factors	Sunlight (vitamin D) significantly affects high-risk gene variants [35].	Not affected by environmental factors.	-
Functional impact	High-risk gene variants target autoimmunity and immune regulation.	There is little information on how low-risk gene variants affect body function.	-
Pathway involvement	High-risk gene variants target cytokine signaling, antigen presentation, and T-cell activation pathways.	Low-risk gene variants primarily target metabolic and neuroprotective pathways. These pathways are not directly linked to MS, which explains the low risk.	-
Gene–gene interactions	Interactions between genes create a combined effect that increases the severity of MS [15].	Rare gene–gene combinations generally have less impact on disease severity.	-
Epigenetic modifications	High-risk gene variants are frequently altered through DNA methylation and histone modifications. These changes affect MS risk and severity.	Research on epigenetic modifications in low-risk rare genes is limited.	-
Gene loci impact	Gene loci associated with high-risk genes affect pathways such as cytokine signaling and T-cell activation.	Gene loci associated with low-risk genes affect neuroprotective pathways.	rs2853035 (in rare variants).
Heritability	A strong family history association exists in high-risk gene variants, indicating that MS can be passed through multiple generations [17].	Rare genes have weak family links and only occur sporadically.	-
Disease severity	High-risk gene variants are associated with aggressive and early-onset forms of MS [8,18].	Disease resulting from low-risk rare gene variants progresses mildly. Severity is also considered as having a role in such scenarios.	-
Response to treatment	Despite being high-risk, common genes have a better response to treatment than rare variants [25]. The main reason for this is that extensive research has been conducted on them.	Diseases resulting from low-risk gene variants typically require personalized treatment solutions because they are not common among the population.	-
Strength of association	There is a strong association between high-risk genes and MS.	There is a weak association between low-risk genes and MS.	-

**Table 4:** Classification of genes assessed in this study.

Gene Name	Role of the Gene in MS Development	Type of Mutation (If Applicable)	Pathway/Mechanism Targeted by the Gene	Gene Risk Classification (Risk of Causing MS)	Associated Gene Loci and Impact
HLA-DRB1 [9,32,36]	The proteins it codes for form part of the myelin-based peptides responsible for autoimmune response. Functional inadequacies could trigger MS symptoms in this manner.	LOF	Immune response regulation	High	The DR15 haplotype regulates antigen–T-cell binding during immune response.
TNFRSF1A [9]	It facilitates inflammatory response in the CNS by activating immune cells that target the myelin sheath, which could cause demyelination and MS symptoms.	REG	Cytokine signaling	High	-
IL-2Rα [15]	It is responsible for T-cell modulation. Gene variation can lead to immune regulation imbalance, which could trigger the T cells to attack the myelin sheath in the CNS, leading to MS symptoms.	LOF	T-cell regulation	High	rs2104282 plays a key role in T-cell differentiation.
IL-1Rα [15]	It could trigger inflammation in the CNS, leading to demyelination and myelin sheath damage.	GOF	T-cell and B-cell signaling	High	-
KIF5A [24]	It is responsible for axonal transport. Mutation in its C-terminal hotspot can result in the formation of classical amyotrophic lateral phenotypes that characterize MS.	REG	Cellular metabolism	Low	Rs123456 plays neuroprotective and axonal repair roles.
REEP1 [24]	It could disrupt communication between the endoplasmic reticulum and mitochondria, which would affect cellular function and cause inflammation.	REG	Neuroprotective pathways	Low	-
CYP27B1 [35]	It is responsible for vitamin D metabolism. Gene variation could cause vitamin D deficiency, thus increasing the risk of MS.	LOF	Immune regulation	Low	-
SLC9A9 [37]	It controls immune cell differentiation and function; hence, variation could induce inflammation and myelin damage.	GOF	Gene expression modulation	High	-
KIR2DL3 [16]	Gene variation could cause inaccurate NK cell modulation, elevating the risk of demyelination.	LOF	Immunological regulation	High	-
ARL17B [16]	It oversees cellular transport processes. Variations could induce unwanted immune responses in these processes, leading to demyelination and a risk of MS.	LOF	Unknown	Low	-
CECR7 [16]	It affects T-cell function, which could cause an attack on the myelin sheaths.	REG	Gene regulation	High	-
CEP78 [16]	Gene variation could cause cellular transport dysfunction, leading to potential demyelination.	LOF	Cellular transport processes	Low	-
IFFO2 [16]	It plays a role in immune system modulation. Variation could induce an inflammatory response, which could attack the myelin sheath.	LOF	Immune regulation	Low	-

**Table 5:** Risk genes and associated MS subtypes.

MS Subtype	Associated Risk Gene/Gene Loci	Mechanisms and Implications
Relapsing-remitting MS (RRMS)	HLA-DRB1*15:01, IL-2Rα	These genes cause T-cell modulation and other immune system activation mechanisms. This process causes the episodes displayed by MS patients.
Primary-progressive MS (PPMS)	KIF5A, REEP1	Rare gene variants such as KIF5A are responsible for axonal integrity, which causes neurodegeneration in PPMS.
Secondary-Progressive MS (SPMS)	TNFRSF1A, HLA alleles	These genes can induce demethylation and chronic inflammation, which often causes patients to transition from RRMS to SPMS.
